# Supplementary material for: Long-read sequencing and genome assembly of natural history collection samples and challenging specimens
Source: Genome Biol. 2025 Feb 10;26:25. doi: 10.1186/s13059-025-03487-9 (PMC11809032; doi:10.1186/s13059-025-03487-9)
Supplement: Supplementary file 3 — Additional file 3: Review history. [file 13059_2025_3487_MOESM3_ESM.docx]

**1^st^ round**

**Reviewer 1**

Bein et al described a strategy to sequence ethanol-preserved samples or samples of low-input DNA with PacBio High-Fidelity (HiFi) reads. The authors produced HiFi reads at various conditions (PCR-free, PCR with PacBio enzymes, PCR with KOD Xtreme Hot Start DNA Polymerase and mixtures of these) for multiple species. They observed improvement in assembly contiguity and completeness, sometimes significantly. A major challenge of long-read sequencing is sample prep given small amount of DNA. The manuscript presents a practical strategy which can be useful for sequencing diverse species.

I don't see major technical issues with the manuscript. I only have a couple of minor suggestions:

* The Data Availability section only mentioned B. torquatus. Will the authors make other samples available?

* The Introduction focused on the DNA extraction of ethanol-preserved samples but many results are about the sequencing of low-input DNA. It would be good to also review the progress on low-input library prep. For example, PMID:30669388 is one of the early papers and may be worth a citation.

**Reviewer 2**

Hiller and colleagues present a very informative report on their experience sequencing and assembling natural history collection samples using PacBio HiFi sequencing along with specialized amplification protocols designed for the recovery of these degraded and difficult-to-sequence samples. The paper is well written, timely, comprehensive, and technically sound. The prospect of generating good-quality genomes for such samples, and the comparative genomic analyses this could enable, is exciting. The authors are clearly experienced in sample handling, sequencing technologies, and bioinformatics analyses and deploy appropriate methods throughout. The manuscript is very nearly publication ready. I have only a few comments that should be addressed in revision, and a few more questions mostly out of curiosity that do not necessarily require revisions.


Major comments:

The contamination issue is a potential serious limitation of sequencing such specimens and should not be underplayed by the manuscript. Some suggestions to better highlight the issue:
-Add the quantitative contamination numbers (which do exist in the supplementary tables) to the main text, rather than using vague phrases like "the vast majority" and "high levels".
-Briefly summarize the types of contaminants found in the main text, and maybe add a "blobtools" figure showing the breakdown of endogenous vs. contaminating contigs in one of the resulting assemblies.
-Add a paragraph to the Discussion on the issue of contamination and any recommended mitigation strategies (either in the lab or informatic processing).

There is a lot of discussion regarding bias in the PCR and HiFi sequencing, but then HiC data is used for the maned sloth without any mention of the sample quality potentially affecting this protocol. I think there have probably been prior studies that looked at this. Can you please add a comment on this and cite any relevant studies showing that HiC on ethanol-preserved samples works OK?

Assembly quality is primarily measured via N50 statistics and gene completeness. It would be good to also show that the amplification protocols do not introduce chimers or other artifacts that affect the structural quality of the resulting assembly. This could be easily done by evaluating the HG002 assemblies for major structural errors by comparing against a reference genome for the same sample (e.g. https://github.com/marbl/hg002).

Figures 1 and 2 reference polymerase C before it is introduced in the text, which I found slightly confusing.

Figure 1 needs a legend describing what each of the line colors/types mean (like Figure 5).

Figure 2 caption should include a brief description of the visualization for those who may not be familiar with the UCSC browser.


Minor comments, mostly out of curiosity:

Do you expect any enrichment for certain types of mutations or sequencing errors in the ethanol-preserved samples, such as the deamination typically seen in ancient DNA? When aligning the HiFi reads against the resulting assembly, do you see a higher-than-usual number of differences between the reads and the assembly consensus sequence, indicating potential random mutations?

Is the time after death known for these samples? Were they immediately preserved? This can also be a big factor in DNA quality, and a comment on recommended sampling strategies might be useful to include (for prospective sampling).

Is there an observable correlation between the age of the samples and the DNA quality? Is there an upper limit on how old a sample can be where this protocol might still work? Some comments in the discussion about what is or is not feasible would be helpful (perhaps combined with the suggested contamination discussion).

**Authors’ response**

Additional analysis conducted outside of reviewer’s requests:

We added an additional analysis to quantitatively substantiate the observations in Figure 2 that polymerase C has a reduced PCR bias compared to polymerase A/B. This analysis is based on comparing normalized read coverage in exonic and repetitive genomic regions and is presented in a new Supplementary Figure 3.

We added to the main text:

“Furthermore, mapping the polymerase C HiFi reads to the C. didactylus assembly covered the regions that completely lacked any read before (Figure 2). Consistent with a higher PCR bias for polymerase A/B, we found that the normalized read coverage in exonic and repeat regions is biased towards a lower coverage for the polymerase A/B data compared to polymerase C to data (Supplementary Figure 3). This confirms that previous read dropouts were not caused by sequence divergence between both sloth species or selective degradation of certain genomic regions in our sample, but by PCR bias associated with the polymerases in the PacBio ultra-low input protocol.”

Reviewer #1:

Bein et al described a strategy to sequence ethanol-preserved samples or samples of low-input DNA with PacBio High-Fidelity (HiFi) reads. The authors produced HiFi reads at various conditions (PCR-free, PCR with PacBio enzymes, PCR with KOD Xtreme Hot Start DNA Polymerase and mixtures of these) for multiple species. They observed improvement in assembly contiguity and completeness, sometimes significantly. A major challenge of long-read sequencing is sample prep given small amount of DNA. The manuscript presents a practical strategy which can be useful for sequencing diverse species.

I don't see major technical issues with the manuscript. I only have a couple of minor suggestions:

We thank the reviewer for these kind words and the useful suggestions.

* The Data Availability section only mentioned B. torquatus. Will the authors make other

samples available?

Yes, all assemblies based on ultra-low input libraries were available for download at https://genome.senckenberg.de/download/GenomesCollectionsPolC. We have now uploaded all raw data and assemblies to NCBI, where they are or will be publicly available soon, and have adjusted the data availability section to

“The raw sequencing data and assemblies for Bradypus torquatus are available under BioProject PRJEB73341 and BioSample SAMEA115348596. An improved version of the Elysia timida assembly after incorporating additional polymerase C reads and Hi-C scaffolding [66] is available under Bioproject PRJNA1119176 and Biosample SAMN42332041. The Scintilla philippinensis assembly and raw sequencing data are available under Bioproject PRJNA1120792. Genome assemblies and raw sequencing data of both catfish genomes are available under Bioproject PRJNA1162287 (Cathorops nuchalis) and PRJNA1162286 (Cathorops wayuu). The Podura aquatica assembly and sequencing data are available under Bioproject PRJNA1163304. Raw reads obtained with polymerase C for HG002 are available on https://downloads.pacbcloud.com/public/revio/2023Q3/KODXtreme/HG002_Twist.target_cov_28_000000.bam. The TOGA annotation for the B. torquatus is available at https://genome.senckenberg.de/download/TOGA/. Ultra-low input based assemblies generated in this study are also available at <https://genome.senckenberg.de/download/GenomesCollectionsPolC>.”

The Introduction focused on the DNA extraction of ethanol-preserved samples but many results are about the sequencing of low-input DNA. It would be good to also review the progress on low-input library prep. For example, PMID:30669388 is one of the early papers and may be worth a citation.

We thank the reviewer for mentioning this and we have extended our explanations to: “For four species (Anguis fragilis, Idiurus macrotis, Cathorops nuchalis, and Cathorops wayuu), the amount of DNA and the DNA fragment sizes were sufficient to prepare an amplification-free PacBio low input library [26] (Supplementary Table 1).”

and in the Methods:

“The low input protocol allows generating PacBio libraries for samples with limited DNA content without amplification [26].”

Furthermore, we now include a new Discussion paragraph on recent methods developments, which highlights potential advantages of the ultra-low input protocols in producing longer reads with very few chimers. We also suggest that further studies are needed to investigate which method works best for specific sample types. The new paragraph is:

“Apart from the ultra-low input protocol, several new approaches have recently been developed to make small amounts of input DNA accessible for long read sequencing. This includes the above-mentioned MDA [41,42,45], adapter ligation via tagmentation [46,47], and Picogram input multimodal sequencing (PiMmS) [48]. We show here that the ultra-low input protocol produces very few chimeric reads in contrast to MDA. Furthermore, the ultra-low input protocol can generate average read lengths of ~10 kb, which is similar to read lengths generated by PiMmS [48], but substantially longer than those generated with tagmentation based approaches (2.5-5 kb averages) [46,47]. Nevertheless, different methods likely have ideal application ranges that depend on the input sample, its quality and amount of DNA. Future research should therefore benchmark which library preparation method is optimal for which sample type. ”

Reviewer #2:

Hiller and colleagues present a very informative report on their experience sequencing and assembling natural history collection samples using PacBio HiFi sequencing along with specialized amplification protocols designed for the recovery of these degraded and difficult-to-sequence samples. The paper is well written, timely, comprehensive, and technically sound.

The prospect of generating good-quality genomes for such samples, and the comparative genomic analyses this could enable, is exciting. The authors are clearly experienced in sample handling, sequencing technologies, and bioinformatics analyses and deploy appropriate methods throughout. The manuscript is very nearly publication ready. I have only a few comments that should be addressed in revision, and a few more questions mostly out of curiosity that do not necessarily require revisions.

We thank the reviewer for these kind words and the helpful comments that we address below.

Major comments:

The contamination issue is a potential serious limitation of sequencing such specimens and should not be underplayed by the manuscript. Some suggestions to better highlight the issue:

-Add the quantitative contamination numbers (which do exist in the supplementary tables) to the main text, rather than using vague phrases like "the vast majority" and "high levels".

We thank the reviewer for highlighting this issue and have now added quantitative contamination numbers and more details to the main text:

“While little contamination was found in the Bradypus torquatus sample (~200 kb mostly assigned to plants), the Anguis fragilis data had higher levels of contamination

(~200 Mb assigned to various bacterial groups), and the vast majority of the sequencing data obtained from the Idiurus macrotis sample were contamination (~75 Mb assigned to various groups of bacteria) (Supplementary Figures 1, 2). High levels of contamination (71-90% of sequenced reads) were also detected for three additional ethanol-preserved samples, where we directly applied the ultra-low input protocol: Russian desman (Desmana moschata) sampled in 1947, Hazel dormouse (Muscardinus avellanarius) sampled in 2016, and a Anguis fragilis sample from 1878 (Supplementary Tables 1, 3). Together, while sample contamination with bacteria, protists and bacterial viruses or cross-contamination with human DNA is another challenge related to samples obtained from collections [33–35], our tests also show…”

Additionally, we added a new column to Supplementary Table 3 to provide more information on the contamination that was found in our samples:

-Briefly summarize the types of contaminants found in the main text, and maybe add a "blobtools" figure showing the breakdown of endogenous vs. contaminating contigs in one of the resulting assemblies.

As mentioned above, we have now added the main sources of contamination in the140

main text. As suggested, we added a new Supplementary Figure 2 showing blobplots for our assemblies:

Supplementary Figure 2: Breakdown of taxa contributing to exogenous DNA contamination.

Blobplots created with blobtoolkit [2] for assemblies created with the ultra-low input protocol. The central plot depicts assembly contigs as blobs, with the blob sizes corresponding to contig sizes (scaled to the largest contig in the assembly) and colors representing the assigned taxon as inferred from the best blast hits. The Y-axis shows the coverage of sequenced reads mapped back to the respective contig and the X-axis shows the average GC-content of each contig. Histograms on top and right hand side summarize average GC-content and coverage of assembly contigs, partitioned by the assigned taxa.

(A) The contig assembly of Idiurus macrotis reads shows that most of the assembled contigs are assigned to different bacteria, and coverage as well as GC content of assembled contigs varies widely, indicating that the assembly mostly consists of contaminant sequence.

(B) For the contig assembly of the Anguis fragilis sample from 2021, the majority of the assembly is assigned to either “Chordata” (dark blue) or “no-hit” (light-blue), and “no-hit” contigs partly resemble “Chordata” contigs in terms of coverage and GC-content. On the other hand, the longest contigs in this assembly are assigned to “Pseudomonadota” (Proteobacteria, orange), meaning that there is evident bacterial contamination present in the assembly. This sample was collected as a roadkill, and partial decomposition by bacteria provides an explanation for the observed contamination.

(C) In the final contig level assembly of Bradypus torquatus, only two small contigs are assigned to “Streptopytha” (green), highlighting the absence of systematic contamination.

(D) The majority of contigs of the Elysia timida hifiasm-phased assembly of haplotype 1 is assigned to “Mollusca” (red), but the blobplot clearly shows several other clusters consisting of large contigs assigned to “Pseudomonadota” (orange) and other bacterial taxa or “no-hit” (light-blue). As the individual sequenced here was immediately killed and homogenized for sequencing without prior fixation, sample decomposition is unlikely. Rather, Elysia timida is too small to dissect before sequencing, therefore the observed contamination is likely caused by the gut microbiome and other commensal microorganisms. It should be noted that mollusca are underrepresented in the blast database, which likely increases false-positive hits and “no-hit” assignments [3].

-Add a paragraph to the Discussion on the issue of contamination and any recommended mitigation strategies (either in the lab or informatic processing).

We fully agree that discussing the problems caused by contamination is important and we have therefore added the following paragraph to the discussion that also emphasizes that importance of removing contamination from the resulting assemblies:

“Contamination caused by sample decomposition, human handlers, or commensal bacteria is expected for collection samples that have been stored under non-sterile conditions [33]. It is difficult to assess contamination prior to sequencing, and we find different levels of contamination in our samples, ranging from most of sequenced reads stemming from contaminants to almost no contamination. Analyzing a low coverage of sequencing reads for contamination before sequencing a sample to the coverage required for assembly could therefore be a cost-efficient strategy to select those samples that contain sufficiently low contamination levels. Furthermore, the resulting assemblies should be carefully screened for contamination using existing methods [43,44].”

There is a lot of discussion regarding bias in the PCR and HiFi sequencing, but then HiC data is used for the maned sloth without any mention of the sample quality potentially affecting this protocol. I think there have probably been prior studies that looked at this. Can you please add a comment on this and cite any relevant studies showing that HiC on ethanol-preserved samples works OK?

We thank the reviewer for raising this point. As mentioned in the manuscript, we used the Arima HiC protocol, which generated useful Hi-C data without obvious issues, which is consistent with results from this publication (http://dx.doi.org/10.1093/gigascience/giac068) and our recent preprint (http://dx.doi.org/10.1101/2024.08.30.610474). Furthermore, in several ongoing genome projects, Arima HiC consistently works for us using ethanol-preserved samples as input material, while we had failures with the OmniC protocol. We do not have systematic benchmarks to draw conclusions on which protocol generally works for ethanol samples; however, we emphasized that Arima HiC worked by adding to the Results and citing these references in

“To obtain a final scaffolded assembly of Bradypus torquatus, we used the Arima HiC protocol, which is applicable to ethanol-preserved samples [23,38], to generate 97.5 Gb in long-range read pair data.”

Assembly quality is primarily measured via N50 statistics and gene completeness. It would be good to also show that the amplification protocols do not introduce chimers or other artifacts that affect the structural quality of the resulting assembly. This could be easily done by evaluating the HG002 assemblies for major structural errors by comparing against a reference genome for the same sample (e.g. https://github.com/marbl/hg002).

We thank the reviewer for this insightful comment, which is highly relevant for the method described here. As suggested, we used the HG002 data to evaluate the number of chimeric reads obtained with polymerase A/B and C. We used non-amplifiied HG002 reads as a baseline. We further included HiFi reads amplified with Multiple Displacement Amplification (MDA), a method known to create many chimeric reads.

Instead of comparing assemblies, we focused on assessing chimers directly in the reads, as this allows us to also quantify low amounts of chimers that will not be introduced in an assembly. To this end, we mapped reads to the HG002 reference genome and determined whether reads have supplementary alignments, which is a sign of chimers.

We found that polymerase C alignments have the lowest fraction of putative chimers (0.54%), very similar to polymerase A/B and non-amplified reads. In contrast, almost 70% of the MDA alignments are likely chimers. We have added these findings as a new results section and a new Supplementary Figure 4.

Results:

“Since PCR amplification may produce chimeric reads [41], we used available non- amplified human HiFi reads produced from the HG002 sample as a baseline to compare the amount of chimeric HiFi reads generated by polymerase A/B and C. We mapped reads to the HG002 assembly [6] and computed the number of reads with supplementary alignments, which indicate chimers. We found that the fraction of chimeric alignments is very low (≤0.81%) across all three libraries, with polymerase C reads having the lowest fraction (Supplementary Figure 4A). We next included available HG002 read data obtained by Multiple Displacement Amplification (MDA) in this comparison. Consistent with previous observations [41,42], the majority of MDA alignments (69.3%) are chimers, which is further supported by the observation that the primary alignment lengths are much shorter than the MDA reads (Supplementary250

Figure 4). We therefore conclude that long range PCR amplification used in the original and modified ultra-low input protocol does not create more chimeric reads than non-amplified libraries and orders of magnitude fewer chimeric reads than MDA libraries.”

Supplementary Figure 4:

Supplementary Figure 4: Low fraction of chimeric reads in amplified PacBio HiFi-read libraries.

(A) The Y-axis shows different types of alignments as classified by minimap2 [4] when mapping human reads to the HG002v1.1 assembly. The X-axis depicts the fraction of alignments from a library. Supplementary alignments of a read indicate that the read is a chimer consisting of two different genomic regions. Alignments of non-amplified ultra-low input reads serve as a baseline and show a similarly low fraction of supplementary alignments (0.72%) as reads obtained with polymerase A/B (0.81%) and polymerase C (0.54%). In stark contrast, the majority of MDA reads have supplementary alignments and are likely chimeric (69.3%).

(B - E) Histograms of raw read and alignment lengths of different PacBio libraries, following the color scheme of A. The filled histogram areas indicate raw read length. The red and blue lines show primary and supplementary alignment lengths excluding soft- and hard-clipped bases. Whereas primary alignment lengths closely follow raw read length distributions for ultra-low and non-amplified reads (B,C,D), indicating full length mapping, for MDA reads (E), the mode of the primary alignment distribution is located at around ~5,000 nt compared to ~11,000 nt length for raw reads, clearly showing that primary alignments tend to be severely truncated. In accordance with (A), the amount and length of supplementary alignments is very low in non-amplified and ultra-low reads, but exceptionally high in MDA reads.

Figures 1 and 2 reference polymerase C before it is introduced in the text, which I found slightly confusing.

We agree that this is confusing as we discuss Figure 2 before explaining the rest of Figure 1. To correct this, we now mention polymerase C early on in the Results in this sentence:

“For simplicity, we refer to these polymerases as “A” and “B” in the following to distinguish them from a third polymerase “C” that we also investigate as described below.”

Figure 1 needs a legend describing what each of the line colors/types mean (like Figure 5).

We agree with the reviewer and have modified the figure legend to provide more explanation.

“The N50 and N90 values are shown as vertical grey lines and indicate contig sizes for which 50% and 90% of the assembly consists of contigs of at least that size, respectively. Assemblies involving polymerase C read data are shown as solid lines, assemblies generated from polymerase A/B data are shown as dashed lines. Colors refer to different comparisons discussed in the text and summarized in the inset.”

The new Figure 1 looks like this:

Figure 2 caption should include a brief description of the visualization for those who may not be familiar with the UCSC browser.

We agree with this suggestion and added to the legend:

“The TOGA gene annotation is shown in blue with boxes representing coding exons, connecting horizontal lines representing introns, and arrowheads indicating the direction of transcription (+ or - strand). Mapped HiFi reads are shown below as boxes with orange tickmarks representing insertions in the B. torquatus reads relative to the C. didactylus assembly. Reads in blue and red align to the + and - strand, respectively.”

Minor comments, mostly out of curiosity:

Do you expect any enrichment for certain types of mutations or sequencing errors in the ethanol-preserved samples, such as the deamination typically seen in ancient DNA? When aligning the HiFi reads against the resulting assembly, do you see a higher-than-usual number of differences between the reads and the assembly consensus sequence, indicating potential random mutations?

When reviewing relevant literature, we found that while deamination is a well-known source of artifact in ancient DNA, there is no evidence for systematically enriched substitutions caused by ethanol preservation (https://www.sciencedirect.com/science/article/abs/pii/S0047248414002619). Irestedt et al. (2022) (https://www.ncbi.nlm.nih.gov/pmc/articles/PMC9542604/) reported deamination from historical DNA samples to occur at the 5’ ends of sequenced DNA fragments, but the insert size of libraries they investigated was well below 1kb. Deamination might therefore also be associated with high rates of DNA fragmentation, which would render a specimen unfit for the sequencing approaches presented in this manuscript.

To ascertain that our samples do not have an excess of deaminated bases at the 5’ ends, we mapped our Bradypus torquatus HiFi reads to the scaffolded and curated B. torquatus assembly and counted substitution patterns at nucleotides 1 - 15 at the beginning of each read and used positions 500 - 1000 as a control. As shown in the figure below, substitution rates are overall very low. Furthermore, substitution rates are slightly higher in internal sections of reads (500 and 1000 bp), providing no indication that deamination events at read beginnings are overrepresented. While there is a slight excess of C->T and G->A substitutions across reads, this pattern has been observed as well in human pseudogenes and mammalian retrotransposons (<https://www.ncbi.nlm.nih.gov/pmc/articles/PMC15487/>; https://www.ncbi.nlm.nih.gov/pmc/articles/PMC203328/) and is therefore more likely a biological signal than a mutational artifact. Hence, we do not find evidence of a systematic pattern of substitutions introduced by ethanol storage.

Is the time after death known for these samples? Were they immediately preserved? This can also be a big factor in DNA quality, and a comment on recommended sampling strategies might be useful to include (for prospective sampling).

Unfortunately time after death is not available for all samples we investigated. Hence, together with other variable aspects of such samples (temperature conditions between death and sampling, details of sample preservation), this increases the uncertainty about whether samples are suitable or not. In our experience, suitability is hard to predict and we typically extract DNA to get a first quick and cheap assessment. For the species, where information for time after death is available, we added this information to the Methods section describing the samples:

“Originally, fin clips of specimens acquired from local fishermen were taken in 2014, immediately placed into ethanol, but subsequently transported multiple times at room temperature until final storage at -20°. The exact time between catch and sampling is unknown, but was likely a few hours.”

“For Elysia timida, we used a whole specimen (~1 cm body length) from our living culture, which we immediately homogenized for DNA extraction after euthanization.”

“For Podura aquatica, we used a single whole specimen (~1.5 mm body length) killed and immediately preserved in 96% ethanol.”

Is there an observable correlation between the age of the samples and the DNA quality? Is there an upper limit on how old a sample can be where this protocol might still work? Some comments in the discussion about what is or is not feasible would be helpful (perhaps combined with the suggested contamination discussion).

In this study, the oldest specimen that we sequenced and assembled into a high-quality assembly was sampled in 2003. From DNA extractions performed on other material not presented here, we have seen that century-old samples can contain DNA fragments of kilobase lengths, rendering them potentially viable for this sequencing protocol. On the other hand, we cannot exclude the possibility that said DNA fragments originate from exogenous DNA contamination rather than endogenous sample DNA. Interestingly, there does not seem to be a straightforward correlation between sample age and DNA quality, likely because other variable factors, such as storage temperature or medium, tissue type and amount of human supervision (for example, how often was the storage medium replenished) also impact sample quality. For example, the Anguis fragilis sample from 2021 was collected only a few years ago, but as roadkill in a state of partial decomposition, leading to DNA fragmentation and contamination. While we were able to sequence endogenous DNA, the output was far below expectations for HiFi sequencing and we found considerable amounts of contamination in the sequenced reads. In their review of sequencing of avian bird skins, Irestedt et al. 2022 state that: “Although the results show that the mapping success to some extent may be correlated with age, we find a comparably high proportion of larger birds (Epimachus) that map poorly, even for the youngest samples”. and “For very old alcohol specimens (preserved before the use of formalin for fixation) we also have a close-to-zero success rate, probably due to hydrolytic damage of DNA when the alcohol concentration has become too low”.

We acknowledge a need to systematically evaluate which factors influence sample quality, and discuss this in a new Discussion paragraph:

“Consistent with previous observations [34], we find that sample age alone is not an accurate predictor of input DNA quality and sample suitability for sequencing. For example, while the B. torquatus sample was collected in 2003, several younger samples exhibited high degrees of DNA degradation (Supplementary Table 1). Hence, in addition to sample age, other factors such as storage temperature and conditions, storage medium, or tissue type likely influence DNA quality. From our experience, samples consistently stored at -20°C and preserved in 96% ethanol perform well, but a systematic assessment of larger sample numbers is needed to substantiate this. ”
